# Spatial distribution of microplastics in Persian Gulf marine environments

**DOI:** 10.1038/s41598-025-31948-5

**Published:** 2025-12-08

**Authors:** Neamatollah Jaafarzadeh, Maryam Ravanbakhsh, Nastaran Talepour, Faezeh Jahedi, Rozhan Feizi, Alireza Reyshahri

**Affiliations:** 1 Environmental Technologies Research Center, Medical Basic Sciences Research Institute, Ahvaz Jundishapur University of Medical Sciences, Ahvaz, Iran; 2 Student Research Committee, Ahvaz Jundishapur University of Medical Sciences, Ahvaz, Iran; 3 Air Pollution and Respiratory Diseases Research Center, Ahvaz Jundishapur University of Medical Sciences, Ahvaz, Iran; 4 Head of Marine Environment Department, Ahvaz, Khuzestan Province Iran

**Keywords:** Microplastic, Marine, Distribution, Inverse distance weighting, Gulf, Ecology, Ecology, Environmental sciences, Ocean sciences

## Abstract

Microplastics (MPs) are recognized as a significant environmental threat within marine ecosystems. This study examines the spatial distribution of microplastics in seawater, sediments, and fish from the Persian Gulf. A total of 24 seawater samples, 24 sediment samples, and 40 specimens of Pennahia anea were collected from eight locations. Samples were digested, and MPs were filtered, counted, and analyzed using a stereo microscope, Raman spectroscopy, and scanning electron microscopy with energy-dispersive X-ray spectroscopy (SEM/EDX). The results revealed the presence of microplastics across all sample types. The total abundance of MPs in seawater ranged from 3 to 15 items/l, in sediments from 10 to 35 items/kg, and in fish from 4 to 18 items/10 g. The study identified a robust positive correlation between microplastic contamination in seawater and fish (*r* = 0.932, *p* = 0.001). Similarly, a strong positive correlation was observed between sediment and fish contamination (*r* = 0.730, *p* = 0.040). In seawater, sediment, and fish samples, microplastics were predominantly fibers, constituting 98.31%, 100%, and 87.5% of the total microplastic content, respectively. These fibers measured ≤ 250 μm in length, with black being the most prevalent colors. The findings highlight that the northern site, Khor Semaili (K1), was a significant MP hotspot. At the same time, areas such as Khor Zangi (K6) and Khor Ghazaleh (K8) exhibited minimal contamination across all matrices. This research highlights the crucial role of spatial analyses in understanding the distribution of microplastics in marine ecosystems.

## Introduction

The proliferation of microplastic (MP) pollution has become a critical environmental issue in the 21 st century, posing significant threats, especially to marine ecosystems^1,2^. MPs, defined as plastic particles smaller than 5 mm, come from different sources^3^. These include the breakdown of larger plastic debris, synthetic fibers from textiles, and microbeads from cosmetics and personal care products^4–6^. Once released into the environment, these particles are highly persistent because of their resistance to degradation^7,8^. Their small size enables them to be easily transported by currents and winds, making MPs ubiquitous in marine ecosystems across the globe^9,10^. The environmental impact of MPs is profound^11,12^. Marine organisms, including plankton and larger fish, can consume these particles. This can cause physical blockages, decreased nutrition intake, and potential exposure to toxic chemicals absorbed onto the surface of microplastics^13^. Furthermore, MPs function as carriers of toxins such as heavy metals and organic pollutants, intensifying health risks for marine organisms and people who consume seafood^7,14^. These pollutants, combined with the long-term persistence of MPs, pose a significant threat to biodiversity and ecosystem health^15^. The environmental impact of microplastics (MPs) is connected to chemicals that adsorb to plastic surfaces or are used in their production. Biologically, microplastics facilitate the geographic transfer of microorganisms, as these organisms can colonize their surfaces and travel with them. Additionally, microplastics pose physical risks to animals through entanglement and ingestion, with entanglement being more common^16^.

The Persian Gulf, characterized by its unique environmental conditions such as high salinity, temperature fluctuations, and its semi-enclosed nature, is particularly vulnerable to MPs pollution^17,18^. As a critical region for global oil extraction, heavy shipping traffic, and rapid coastal urbanization, the Gulf is a hotspot for anthropogenic pollution, including plastic waste. MPs in this region not only threaten the rich marine biodiversity but also raise concerns about human health, given the Gulf’s importance as a fishing region^19,20^. Recent studies have found MPs in the Gulf’s seawater, sediments, and marine organisms. This emphasizes the necessity for thorough spatial analysis to gain a better understanding of the distribution and concentration of these pollutants^21–25^. However, the spatial variability and potential transport pathways of MPs in the Gulf remain understudied, making it difficult to target specific areas for mitigation.

This study focuses on examining the spatial patterns of MPs in seawater, sediments, and fish at eight distinct locations in the Gulf. Using the Inverse Distance Weighting (IDW) interpolation method, a geostatistical technique that considers spatial correlation, we can predict MPs concentrations in unsampled areas^26^. IDW is an effective tool in environmental studies for creating accurate spatial predictions and identifying pollution hotspots, where further management efforts can be concentrated^27,28^. This research will provide crucial insights for policymakers and environmental managers, helping address the problem of MPs pollution in marine ecosystems at both local and global levels.

Mapping the spatial variability of MPs in the Gulf aims to provide a clearer understanding of their transport and accumulation patterns. These findings will contribute to the literature on MPs pollution and provide a foundation for future research on reducing MPs contamination in marine environments. This will help protect the health of ecosystems and human populations.

## Materials and methods

### Study area

**Fig. 1:**
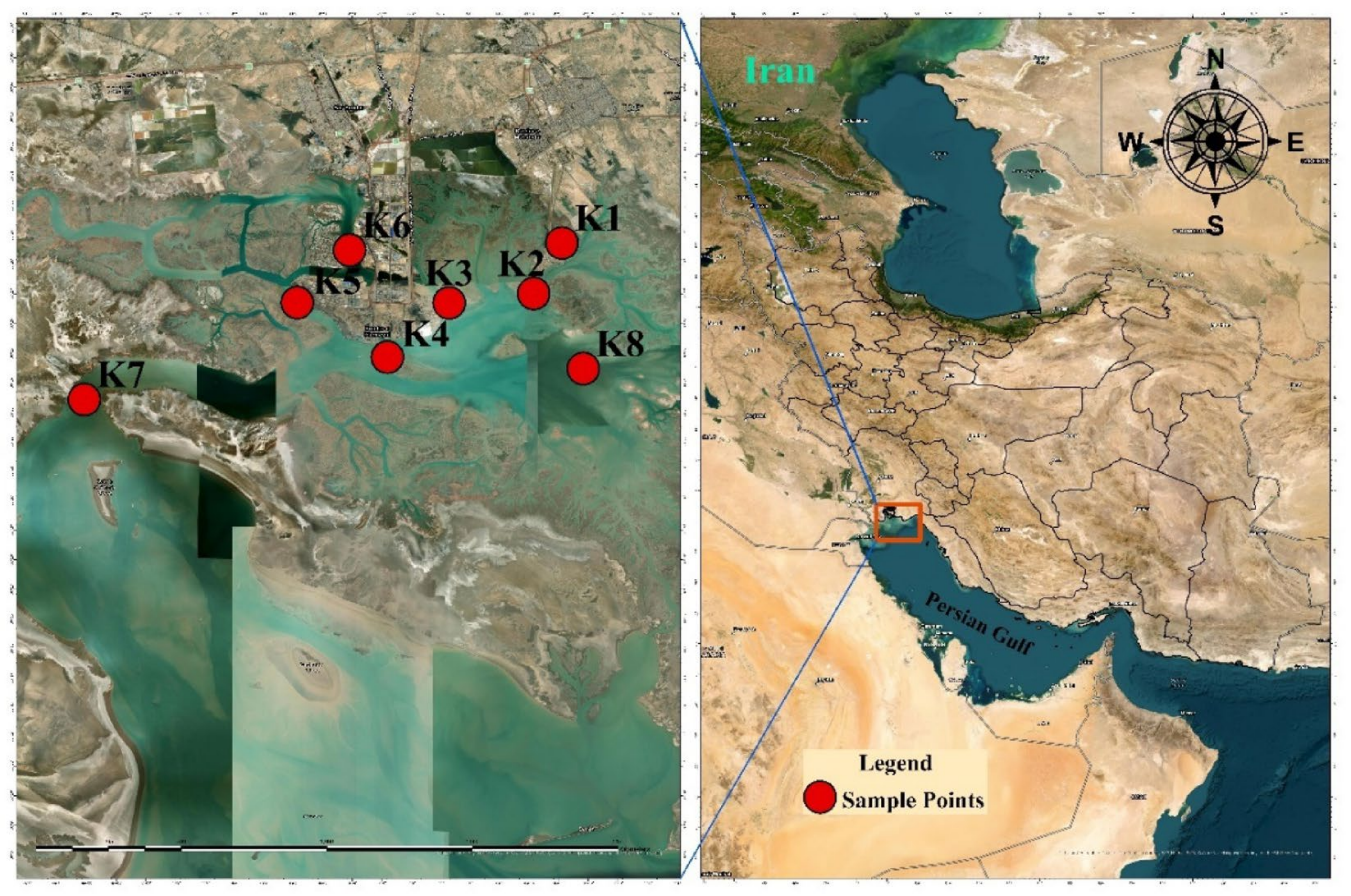
Study area and sampling points (The map was created using ArcGIS Pro version 3.4.0).

The study was carried out in the Musa Estuary, located on the northern coast of the Persian Gulf in Iran. The strategic selection of eight sampling sites is represented in Fig. 1, which includes Khor Bihad, Khor Douragh, Khor Ghazaleh, Khor Semaili, Khor Majidiye, Khor Marimous, Khor Zangi, and Khor Jaafari. These locations were carefully chosen to include a diverse range of environments and potential microplastic pollution origins. The sampling points were chosen based on their proximity to industrial areas, urban centers, and the protected Hara Forest region. The establishment of eight sampling zones was a consequence, with each zone being approximately 3.5 to 4 km apart, across a 75 km² estuarine area.

### Sampling method

Seawater, sea sediments, and fish samples were collected in various zones during September 2022. Seawater and sediment samples were gathered within each zone, with approximately 300 to 400 m between intervals, and at least three subsamples per site. The subsamples were thoroughly mixed, creating a composite sample and minimizing random variation. The preserved samples were stored in glass containers that were pre-cleaned. Calculation of the sample sizes involved the use of Eq. 1.

1 $$\:n={\left(\frac{{Z}_{\left(1-\frac{\propto\:}{2}\right)}\times\:SD}{d}\right)}^{2}$$

The sample size, represented as n, is determined with a 99% confidence level, using a Z-value of 1.96 and a margin of error of 0.1 µ (mean value)^29^.

Table 1 provides a detailed description of the sampling zones.

**Table 1:** Sampling zones in the study area.

Zones	Sub-estuary	Latitude	Longitude	Description	Number of Samples		Fish
					Seawater	Sediment	
K1	Khor Semaili	30.478775°	49.185151°	The harbor in Mahshahr City has facilities for docking fishing vessels and petroleum storage tanks.	3	3	5
K2	Khor Majidiye	30.450252°	49.169189°	Location with major oil infrastructure, global oil export hub, and key transport channel.	3	3	5
K3	Khor Jaafari	30.444906°	49.121440°	Release site for municipal, industrial, and petrochemical wastewater.	3	3	5
K4	Khor Marimous	30.414294°	49.086511°	Port for petrochemical export operations.	3	3	5
K5	Khor Douragh	30.445049°	49.035598°	International port at IKH.	3	3	5
K6	Khor Zangi	30.474721°	49.065155°	Water route designated for fishing boats.	3	3	5
K7	Khor Bihad	30.390335°	48.915232°	The shipping route for oil tankers and vessels, connecting the Bihad area to the Musa estuary.	3	3	5
K8	Khor Ghazaleh	30.407801°	49.197258°	Recreational zones have minimal direct impact from pollution sources.	3	3	5

### Sample preparation

#### Seawater

At depths spanning from 0.5 to 1 m, a total of 24 samples were collected, accumulating to 10 L in volume. To separate large debris and retain microplastic particles, seawater samples were filtered using stainless steel mesh sieves with a pore size of 200 μm. Clean, labeled glass containers were used to store the samples at 4 °C before they were transported to the laboratory. Each sample was treated with 5 mL of 30% hydrogen peroxide for 10 days until no more bubbles were observed to degrade organic matter. Next, the sample was exposed to a Zinc chloride solution (ZnCl_2_) with a density of 1.6 g/cm³, enabling the collection of floating microplastics. The filtrate was subjected to vacuum filtration using filter papers (grade 589/3blueribbon, pore size < 2 mm). After air-drying, the filters were transferred to the Petri dishes for additional analysis^30–32^.

#### Sediment

A total of 24 sediment samples were collected using a grab sampler from the top 0–10 cm layer, and a 200 g subsample from each was sieved to isolate microplastics (MPs).Glass jars containing wet sediments were dried in an oven at 40 °C^33^. After drying, the samples were passed through a 5 mm metal sieve and transferred into glass beakers, which were maintained at room temperature. To prevent the misidentification of organic matter as microplastics MPs and minimize the risk of overestimating their abundance or dimensions, a digestion process was applied. This step was carried out according to the procedure outlined in^34^. The oxidation of organic matter in the sediment was achieved by using 30% hydrogen peroxide (H2O2) for 10 days, until no further bubble formation occurred, indicating complete oxidation. The remaining H_2_O_2_ was poured off, and the sediment was washed with deionized water to eliminate any remaining H_2_O_2_ and gather particles stuck to the beaker walls. The solutions were given several minutes for suspended particles, including MPs larger than 100 μm, to settle. The extraction of MPs particles from sediment samples involved flotation in a saturated ZnCl_2_ solution (density: 1.6 g/cm³)^35^. The separation of lighter MP particles from denser mineral particles was made possible by this process. The supernatant from both the treated sediment and water samples underwent centrifugation at 4000 rpm before being filtered using a (grade 589/3blueribbon, pore size < 2 mm) with the help of a vacuum pump. Following the drying process, the filters containing MPs particles were transferred to Petri dishes for additional investigation^35^.

#### Fish

A total of 40 individuals of Pennahia anea were obtained from local fisheries, and their digestive tracts were analyzed for MPs. This species was selected due to its ecological representativeness, commercial importance, and direct exposure to both pelagic and benthic microplastic sources^36^. The samples were quickly taken to the lab in insulated coolers with ice and kept in a freezer at − 20 °C for future analysis. The tissue samples underwent a thorough rinsing with distilled water, followed by cutting them into approximately 10 mm cubes using pre-cleaned scissors, and finally homogenizing them with a laboratory spatula. Each wet tissue sample, weighing about 10 g (± 0.1 g), was placed in a separate 150 mL glass beaker that had been pre-cleaned. Approximately 40 mL of a 10% KOH solution was added to each beaker. The beakers were covered in aluminum foil and placed in a preheated oven at 40 °C for 72 h, with manual shaking every 24 h (Daniel et al., 2020). 30 mL of ZnCl_2_ solution (density: 1.6 g/cm³) were added to the H_2_O_2_-digested samples to separate microplastics from residual materials. The mixture was centrifuged at 300 rpm for 3 min and then filtered using a vacuum pump and a 10-micron pore-size filter paper. To prevent filters from getting clogged by incompletely digested gills and skin, a two-step filtration process was implemented. At first, a stainless-steel sieve (1000 μm) was utilized to strain out larger particles. The collected materials, including fish scales and bones, were moved to a cleaned petri dish for microscopic analysis. The filtrate was subjected to vacuum filtration using filter papers (grade 589/3blueribbon, pore size < 2 mm). After air-drying, the filters were transferred to the Petri dishes for additional analysis^37^.

### MPs particle identification

Each filter had visually identifiable MP particles. Using a stereo microscope (Sairan-ZSM-1001), the researchers examined key characteristics such as size, shape, and color at magnifications of up to 200 ×^38^. MPs particles were classified by their length (L) into four different size ranges: 100 ≤ L < 250 μm, 250 ≤ L < 500 μm, 500 ≤ L < 1000 μm, and 1000 ≤ L < 5000 μm. The particles were categorized based on their shape as fibers, fragments, and pellets, and based on their color into five groups: black, blue, yellow, redand whiteransparent^35^.

The Scanning Electron Microscope (SEM, TESCAN Vega 3, Czech Republic) with a 2 nm resolution at 20 kV was used to analyze the surface morphology and elemental composition of MP particles. It was equipped with an energy-dispersive X-ray microanalyzer (EDS). To conduct SEM-EDS analysis, MP particles were placed on copper adhesive tape and coated with gold. The polymeric composition of the MPs was determined by employing a confocal Raman microscope (Lab Ram HR Evolution, Horiba Japan) with a 785 nm laser, detecting between 400 and 1800 cm⁻¹. The reference spectra were used to match the MPs particle spectra and identify the polymer type. Polymeric materials are often characterized using Raman spectroscopy^39,40^.

### Ecological risk assessment

To quantify the level of microplastic contamination across different environmental matrices, the Pollution Load Index (PLI) was calculated for seawater, sediment, and fish samples. The PLI for each matrix was determined using the formulas (2 and 3):

2 $$\:PLI=\raisebox{1ex}{${C}_{i}$}\!\left/\:\!\raisebox{-1ex}{${C}_{0}$}\right.$$

3 $$\:{PLI}_{Zone}=\sqrt[n]{{PLI}_{1}\times\:{PLI}_{2}\times\:\dots\:\times\:{PLI}_{n}}$$

where C_i_ represents the total concentration of microplastics at each sampling site, and C_0_ corresponds to the minimum observed microplastic concentration within the respective matrix; n represents the total number of sampling points, and PLI_Zone_ denotes the overall Pollution Load Index of microplastics calculated for the zone^41,42^.

### Quality assurance and quality control (QA/QC)

Comprehensive quality assurance and quality control (QA/QC) measures were implemented throughout the sampling, extraction, and laboratory procedures to ensure the integrity of the results. Glass containers used at various stages were pre-treated by immersion in 10% nitric acid (HNO₃) for 24 h, followed by thorough rinsing with filtered distilled water to remove potential contaminants. Laboratory surfaces were sanitized with ethanol to maintain a clean working environment. To avoid microplastic contamination, only metal and glass containers were employed throughout all steps. Additionally, doors and windows were kept closed to minimize airborne contaminants. Personnel conducting the experiments wore white cotton lab coats, disposable latex gloves, and facemasks to prevent cross-contamination. All solutions and reagents were pre-filtered through 1–5-micron filter paper to remove particulates. As a further precaution, control samples (blank tests) accompanied the experimental samples throughout the analytical process to detect any potential contamination from laboratory materials or reagents. No polymer traces were found in these control samples.

### Inverse distance weighting (IDW)

Inverse Distance Weighting (IDW) is a widely utilized interpolation technique in environmental studies to estimate unknown values based on measured data points. In this study, the IDW in ArcGIS Pro (version 3.4.0) was employed to model the spatial distribution of microplastics (MPs) concentrations across three environmental matrices—seawater, sea sediment, and marine organisms—at eight sampling points within the Gulf. This interpolation method was selected due to its ability to account for the spatial influence of neighboring points, ensuring that closer samples have a greater influence on predicted values, which aligns well with the localized nature of MPs pollution.

IDW assumes that the influence of a given data point diminishes with distance. Therefore, the estimated MP concentrations at unsampled locations were generated through a weighted average of known concentrations, with weights inversely proportional to the square of the distance. The method ensures that the maximum and minimum values of the interpolated surface occur only at the sample points, maintaining the integrity of our measured MPs concentrations.

This spatial analysis will help identify potential MPs accumulation hotspots across the Gulf, supporting targeted mitigation efforts. Additionally, IDW provides insights into the variability between different matrices—water, sediment, and marine organisms—enabling comparisons of accumulation patterns. IDW interpolation does not introduce assumptions beyond spatial proximity, so it offers a reliable and straightforward approach to visualize and predict MP concentrations in the study region^43^.

## Results and discussion

### Abundance and distribution of microplastics

**Fig. 2:**
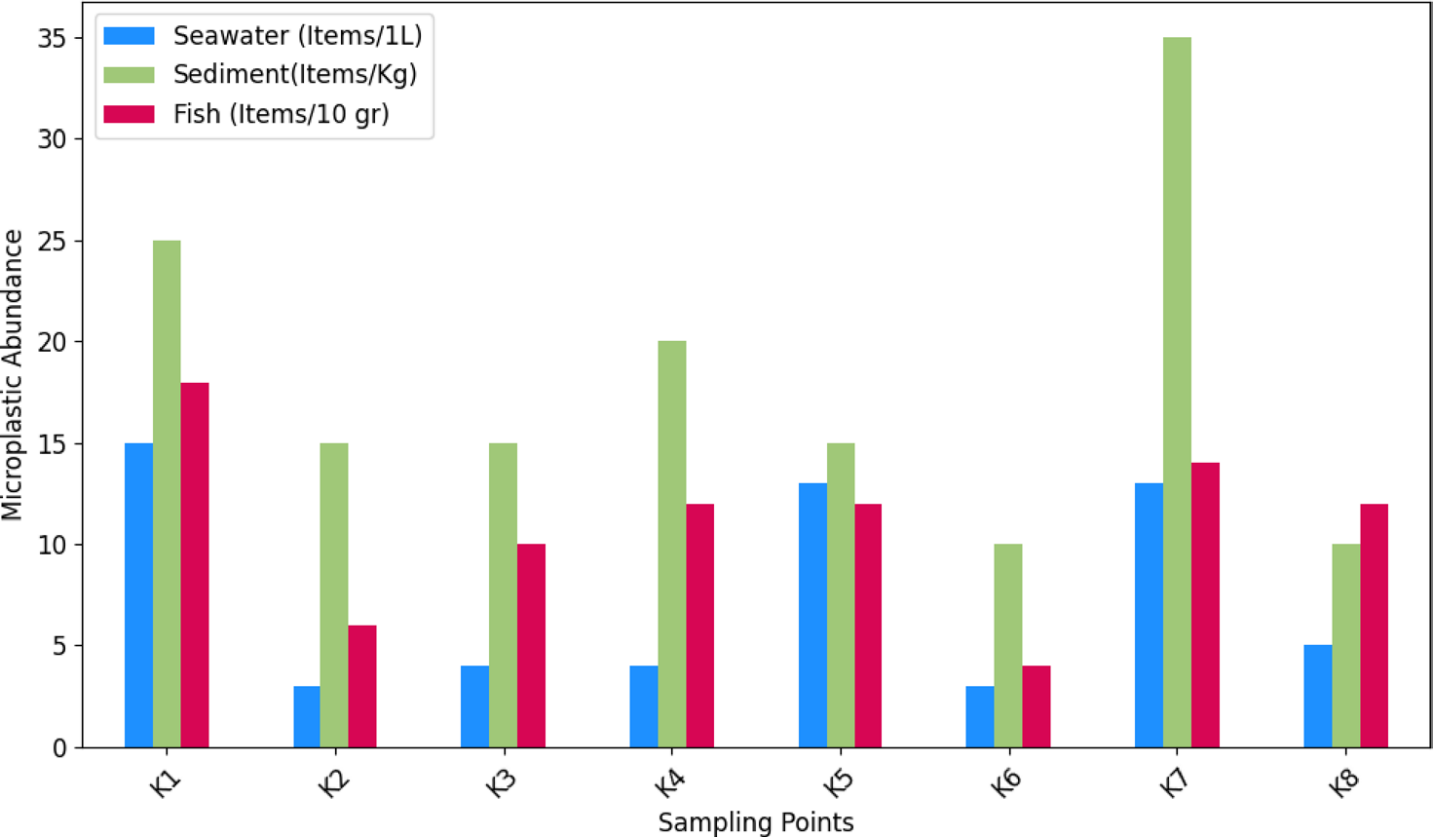
Comparison of microplastic abundances across various sampling points.

The study verified the presence of microplastics in all examined samples, encompassing seawater, sediments, and marine fish from the Gulf. The concentration of microplastics in the analyzed samples ranged from 3 to 15 items l^− 1^ in seawater, 10 to 35 items Kg^− 1^ in sediments, and 4 to 18 items 10 g^− 1^ in fish (Fig. 2). These values represent the range of MP concentrations observed among individual samples. In total, 60 items L^− 1^ were detected in seawater, 145 items kg^− 1^ in sediments, and 88 items 10 g^− 1^ in fish, reflecting the cumulative number of MP particles identified across all analyzed samples. The Kolmogorov–Smirnov test revealed that the abundances of microplastics across all samples exhibited a non-normal distribution. Spearman’s correlation analysis reveals varying degrees of association between microplastic concentrations in seawater, sediment, and fish samples. This study demonstrated a strong positive correlation between microplastics (MPs) contamination in seawater and fish (*r* = 0.932, *p* = 0.001), highlighting that higher MPs levels in water directly increase contamination in fish. A similarly significant positive correlation was found between sediment and fish contamination (*r* = 0.730, *p* = 0.040), suggesting that sediment-bound microplastics may contribute to fish exposure. In contrast, the correlation between seawater and sediment (*r* = 0.569) was moderate and not statistically significant (*p* = 0.141), indicating an inconsistent connection between these two environmental matrices.

These findings partially align with those of Zhang et al., who observed a positive correlation between fish and sediment MPs, though they noted variations based on particle shape and color. However, they did not find any correlation between fish and water MPs^44^. Similarly, Agharokh et al^45^. and Yuan et al^46^. reported no significant relationship between MP levels in surface water and sediment. Gao et al. observed a negative correlation between pelagic fish ingestion and MP concentrations in surface water, whereas benthic fish exhibited a positive correlation with sediment MPs. Interestingly, Gao et al. also identified a perfect negative correlation between pelagic fish and benthic MPs, contrasting with the positive relationship found between MPs in surface and benthic^47^.

The non-normal distribution of microplastic abundance suggests that their dispersion is heterogeneous, likely influenced by local pollution sources, hydrodynamics, and sediment dynamics. Notably, the strong positive correlation between seawater and fish contamination underscores that marine organisms are directly affected by microplastics present in the water column. This relationship highlights the potential risks to marine biodiversity and food safety, as fish contaminated with microplastics may enter the human food chain^47–49^.

Furthermore, the correlation between sediment and fish contamination suggests that sediments may serve as reservoirs for microplastics, subsequently affecting both benthic and pelagic organisms. This finding aligns with existing literature, which emphasizes the role of sediments in microplastic retention and their transfer to higher trophic levels. Studies investigating the toxic effects of microplastics on aquatic organisms consistently demonstrate alterations in biochemical and behavioral characteristics among exposed species. Additionally, exposure to microplastics has been associated with increased mortality. As ingested microplastic particles travel up the food chain, they have the potential to reach higher trophic levels, posing significant ecological and health risks^50–54^.

Although in this study, there was no significant correlation between sampling points, microplastic concentrations in seawater, sediments, and fish vary significantly across different zones. Similarly, Agharokh et al. investigated the relationship between microplastic (MP) abundance across sediments, surface waters, and fish along the Gulf. Their findings revealed no correlation between sampling sites for sediments, surface waters, and fish species^45^. In seawater, the highest concentration is observed in Khor Semaili (K1), with 15 items per liter, while Khor Majidiye (K2) and Khor Zangi (K6) report the lowest at 3 items per liter. The average concentration across all zones is approximately 7.5 items per liter. In sediments, a broader range is evident, with a maximum of 35 items per kilogram found in Khor Bihad (K7) and Khor Zangi (K6), reporting the least at 10 items per kilogram; the overall average concentration in sediments is around 20 items per kilogram. Regarding fish samples, Khor Semaili (K1) shows the highest contamination level at 18 items per 10 g, whereas Khor Zangi (K6) exhibits the lowest contamination at 4 items per 10 g, with an average concentration of about 10.75 items per 10 g. Both Khor Semaili (K1) and Khor Bihad (K7) demonstrate relatively high levels of microplastics in seawater and fish, suggesting potential local pollution sources. Conversely, Khor Zangi (K6) consistently displays the lowest levels across all categories, indicating it may be less affected by microplastic pollution. These concerning levels, particularly in sediments and fish, raise implications for marine ecosystems and food safety, highlighting those certain zones, especially Khor Semaili (K1) and Khor Bihad (K7) are significantly impacted by microplastic contamination.

### Morphological characteristics of microplastics

**Fig. 3:**
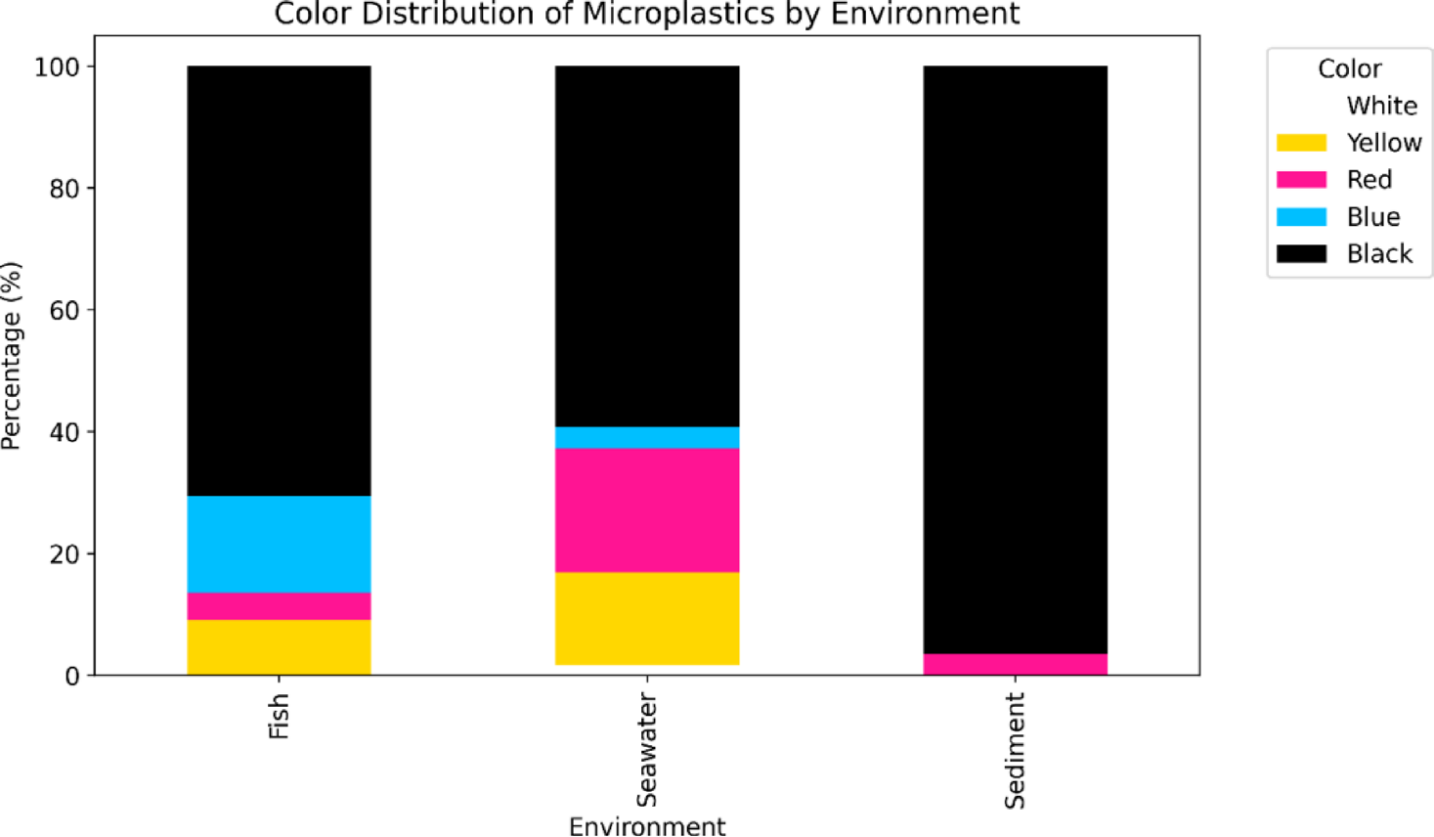
MPs distribution by color in seawater, sediment, and fish samples.

Notable differences in the color distribution of microplastics were observed across different environmental matrices.(Fig. 3). In seawater samples, blackmicroplastics dominate, comprising 59.32% of the total, which indicates significant pollution or fragmentation; these are followed by red (20.34%) and yellow (15.25%). Blue microplastics, on the other hand, constitute only 3.39%, representing a smaller fraction. In sediment samples, black microplastics overwhelmingly prevail at 96.55%, suggesting a substantial local source of microplastics, with redpink microplastics accounting for just 3.45%, indicating limited color diversity compared to seawater. Moreover, the microplastics ingested by fish primarily consist of black (77.5%) and red(5%), while blue microplastics (17.5%) show some variation in the types consumed.

In this study, fiber MPs were dominant in the three media, which was consistent with the results of previous research in the Persian Gulf^32,37,45^.

The predominance of black microplastics in seawater (59.32%) and sediments (96.55%) is alarming and indicative of substantial pollution sources, possibly linked to urban runoff, industrial discharges, petroleum‑derived residues, or the degradation of larger plastic debris. This coloration may reflect the use of carbon-based additives commonly found in plastic products, which not only facilitates identification but also serves as an indicator of microplastic age and degradation state^55^. Progressive color fading, surface dullness, and pigment bleaching generally occur during photooxidation, ultraviolet (UV) exposure, and hydrocarbon-associated weathering, leading to polymer chain scission, oxidation, and biofouling-related discoloration. Petroleum-based plastics and oil residues are known to contribute dark-coloured microplastics due to carbon-rich compounds and accelerated weathering in marine environments, particularly near harbours, fuel terminals, and fishing zones^56,57^. Such alterations are widely recognized as reliable markers of weathering intensity and environmental residence time of microplastics^58,59^.

The relatively low occurrence of blue microplastics, particularly in sediment samples (only 3.45%), could suggest that these colors are less frequently produced or more readily degraded in the environment^60^.

Furthermore, the color distribution observed in fish samples indicates a dietary preference or ecological availability, with a significant 77.5% of ingested microplastics being black. This raises concerns regarding bioaccumulation and potential toxicity, as darker plastics may possess higher levels of absorbed pollutants or additives, which can be transferred through the food chain, thereby affecting higher trophic levels.

**Fig. 4:**
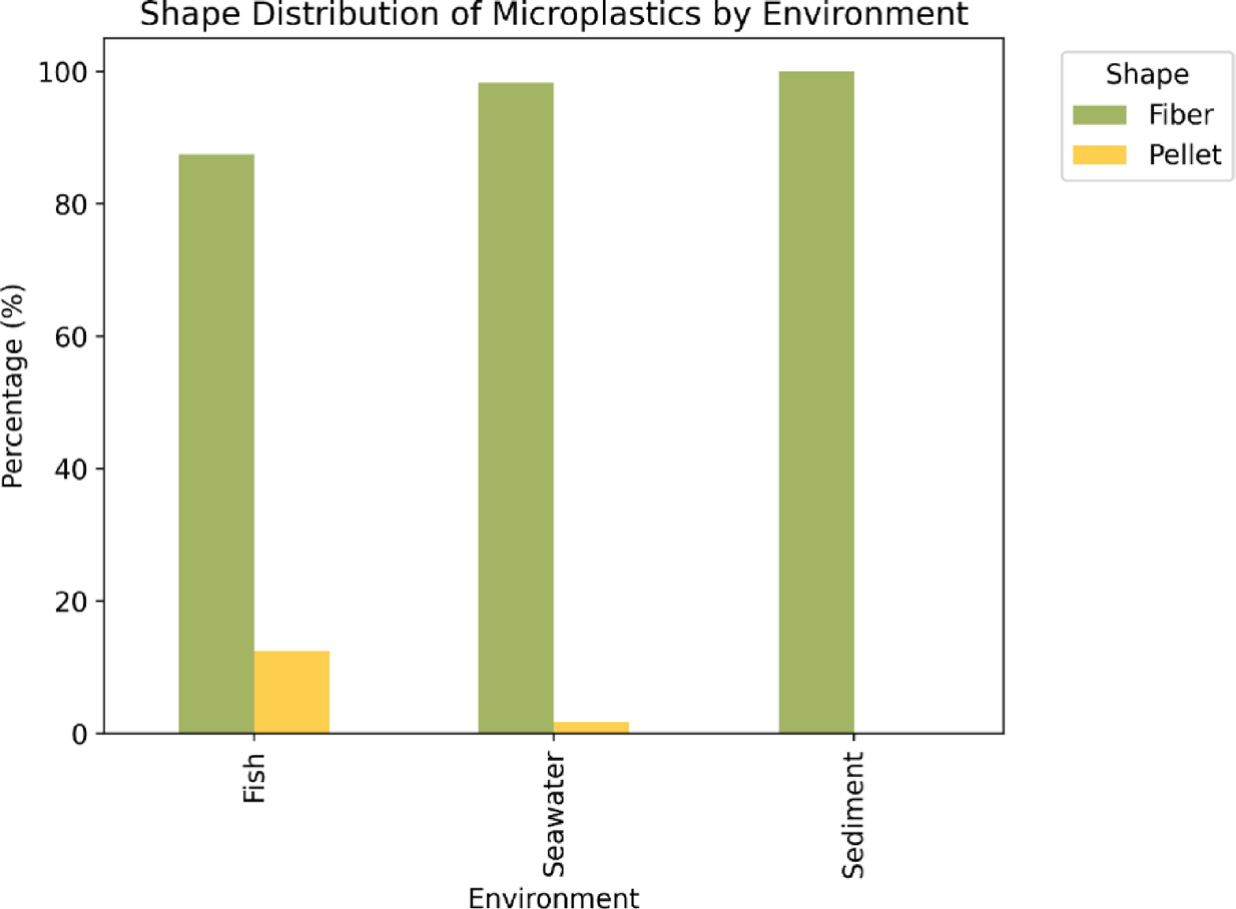
MPs distribution by shape in seawater, sediment, and fish samples.

The analysis of microplastics in various environments revealed significant variations in shape distribution (Fig. 4). In seawater, fibers constituted the dominant morphological category, accounting for 98.31% of all detected microplastics, underscoring the prevalence of filamentous plastics in marine environments. Pellets represented a minor fraction (1.69%), suggesting their limited contribution to the total microplastic load. In sediments, fibers were the only shape observed (100%), confirming their overwhelming dominance in benthic environments. Similarly, in fish, fibers made up 87.5% of ingested microplastics, while pellets accounted for the remaining 12.5%. The overwhelming prevalence of fibers across all sample types indicates that filamentous microplastics are the primary form entering and persisting within these coastal ecosystems. This predominance is likely linked to intensive fishing activities and urban influences in the sampling areas, where sources such as fishing nets, ropes, and wastewater effluents release substantial amounts of fibrous materials. These findings are consistent with previous studies in the Gulf, which have likewise reported fibers as the dominant microplastic form^61^.

The notable abundance of fibers found in this study is largely due to fishing gear such as ropes and nets,, as well as other anthropogenic sources, including textiles, wastewater, and urban runoff, reflecting the multiple pathways through which microplastic fibers enter the marine environment^62^. This observation is consistent with several previous studies in the Persian Gulf, which have also identified fibers as the predominant type of microplastics in these waters^32,37,45^.

Fiber dominance has profound implications for aquatic life, especially for filter feeders and species that mistake these particles for food. The high ingestion rate of fibers (87.5% in fish) raises concerns about potential physiological impacts on fish, including digestive blockages or alterations in feeding behavior, which could have cascading effects on fish populations and the broader ecosystem^48^.

**Fig. 5:**
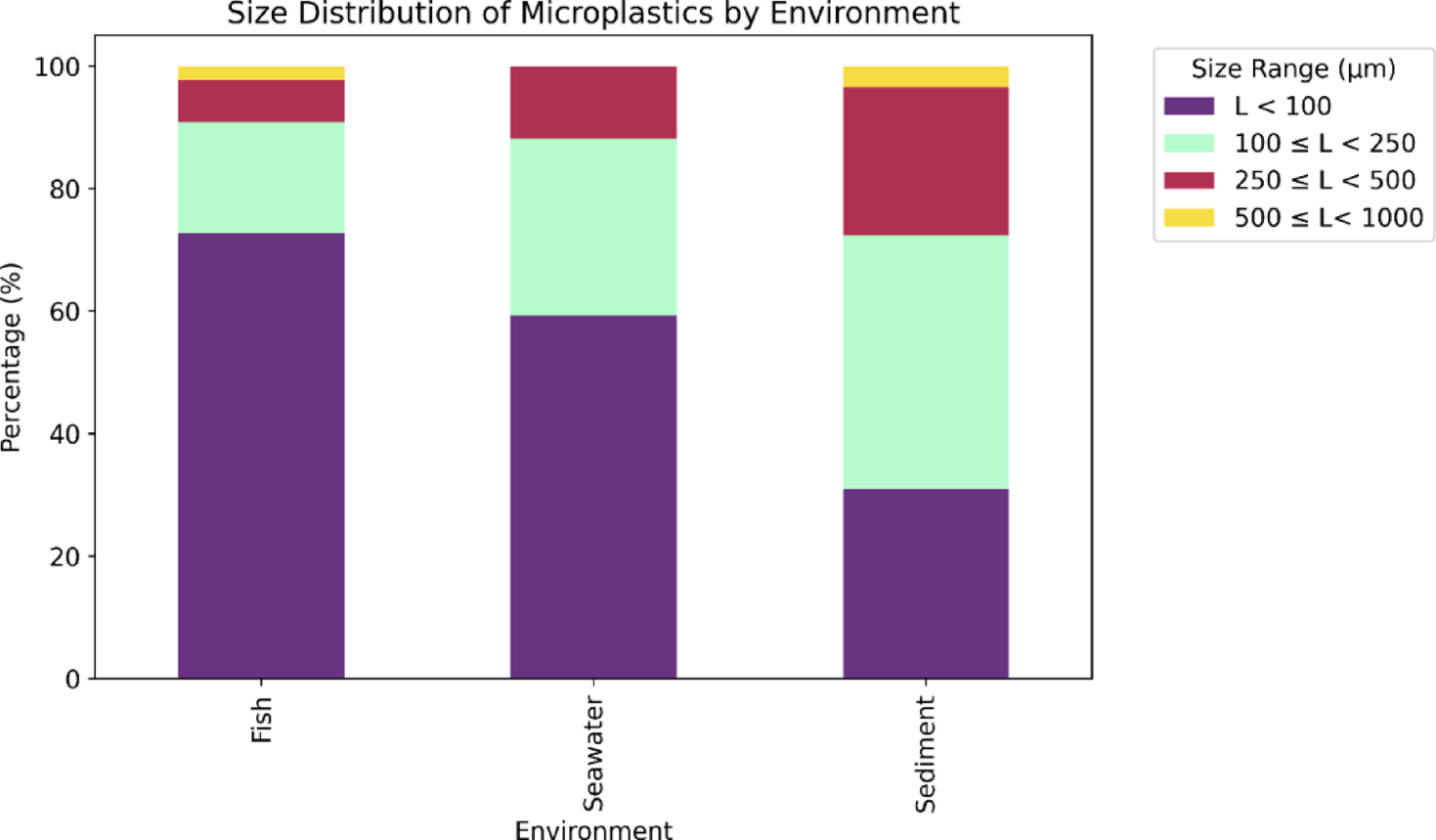
MPs distribution by size in seawater, sediment, and fish samples.

The examination of microplastics in various settings showed significant differences in their size distribution (Fig. 5). In seawater, microplastics smaller than 100 μm constituted the majority (59.32%), indicating a strong tendency for finer particles to remain suspended. Particles between 100 and 250 μm accounted for 28.81%, while those ranging from 250 to 500 μm were much less frequent (11.86%), reflecting a scarcity of larger items. In sediments, particles sized 100–250 μm were relatively dominant (41.38%), followed by smaller ones (< 100 μm, 31.03%), whereas in fish, microplastics < 100 μm prevailed (72.73%), suggesting selective ingestion of finer particles. Small microplastics commonly originate from the progressive breakdown of larger plastics through mechanical abrasion and photodegradation^63^.

Beyond size variability, morphological composition also revealed an absence of fragments in the analyzed samples. This lack of fragments is plausibly linked to the predominance of very small particles in our dataset. Given that fragments typically derive from the disintegration of larger debris and are more frequently detected at sizes above 250 μm, the scarcity of particles exceeding this threshold likely constrained their occurrence. Additionally, the sampling sites were subject to intense fishing and urban activities, environments where fibrous and pellet-type microplastics are more abundant, whereas larger debris capable of generating fragments are relatively scarce.The observed size distribution provides critical insights into the environmental behavior and bioavailability of microplastics. The predominance of particles smaller than 100 μm in seawater and fish suggests that finer microplastics remain suspended longer in the water column, increasing their potential for uptake by diverse organisms^51,64^.

In contrast, the relatively higher proportion of 100–250 μm particles in sediments reflects the sedimentation of denser or aggregated microplastics. Although smaller particles are less abundant in sediments, their presence remains ecologically significant, as benthic organisms may ingest them, potentially facilitating the transfer of microplastics through sedimentary food webs^50^.

**Fig. 6:**
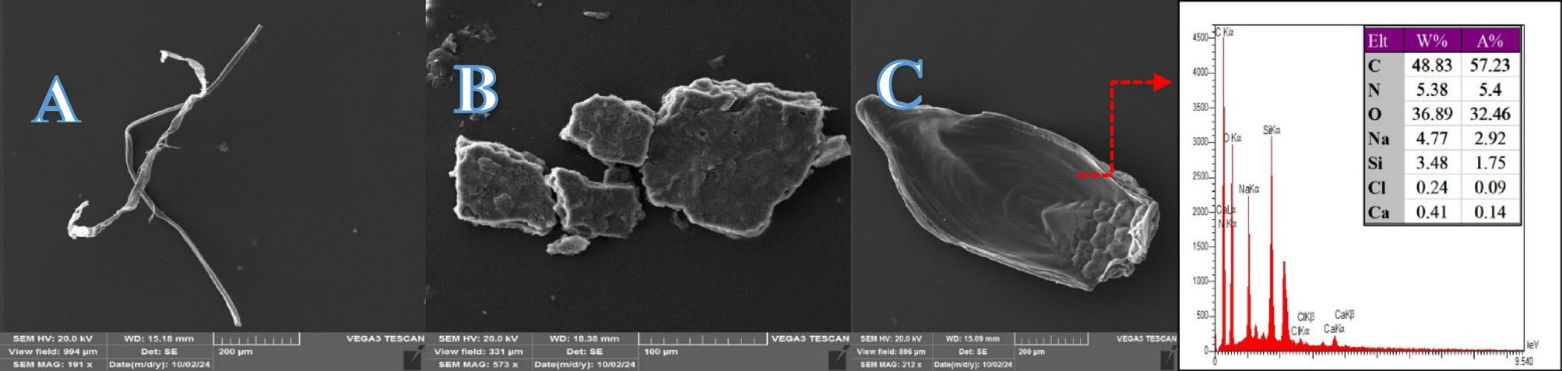
Identification of microplastic particles using SEM/EDS.

The SEM/EDS showcases the surface morphology and elemental composition of microplastics. Scanning electron microscopy (SEM) images show a diverse array of particle sizes and shapes, exhibiting unique characteristics that reflect weathering and fragmentation processes typically linked to environmental degradation. One representative sample was selected for EDX analysis from the identified microplastic samples, which serves as a limitation of this study (Fig. 6). The microplastic samples analyzed using SEM-EDS revealed several elemental compositions indicative of polymer types and environmental interactions. Carbon (C), which comprised 48.83% of the weight, confirmed the synthetic polymer nature of the material, suggesting the presence of polymers like polyethylene (PE) or polypropylene (PP). Oxygen (O), contributing 36.89%, hinted at either oxidative degradation or polymers containing oxygen, such as polyethylene terephthalate (PET). The nitrogen (N) content of 5.38% indicated the possible presence of polyamide (nylon) or contamination from biological sources. Trace elements such as sodium (Na) (4.77%) and silicon (Si) (3.48%) reflected environmental exposure, possibly from seawater or contact with silicate materials. Calcium (Ca) (0.41%) suggested mineral deposits or fillers, while the minor presence of chlorine (Cl) (0.24%) pointed toward potential contamination or the presence of polyvinyl chloride (PVC).

**Fig. 7:**
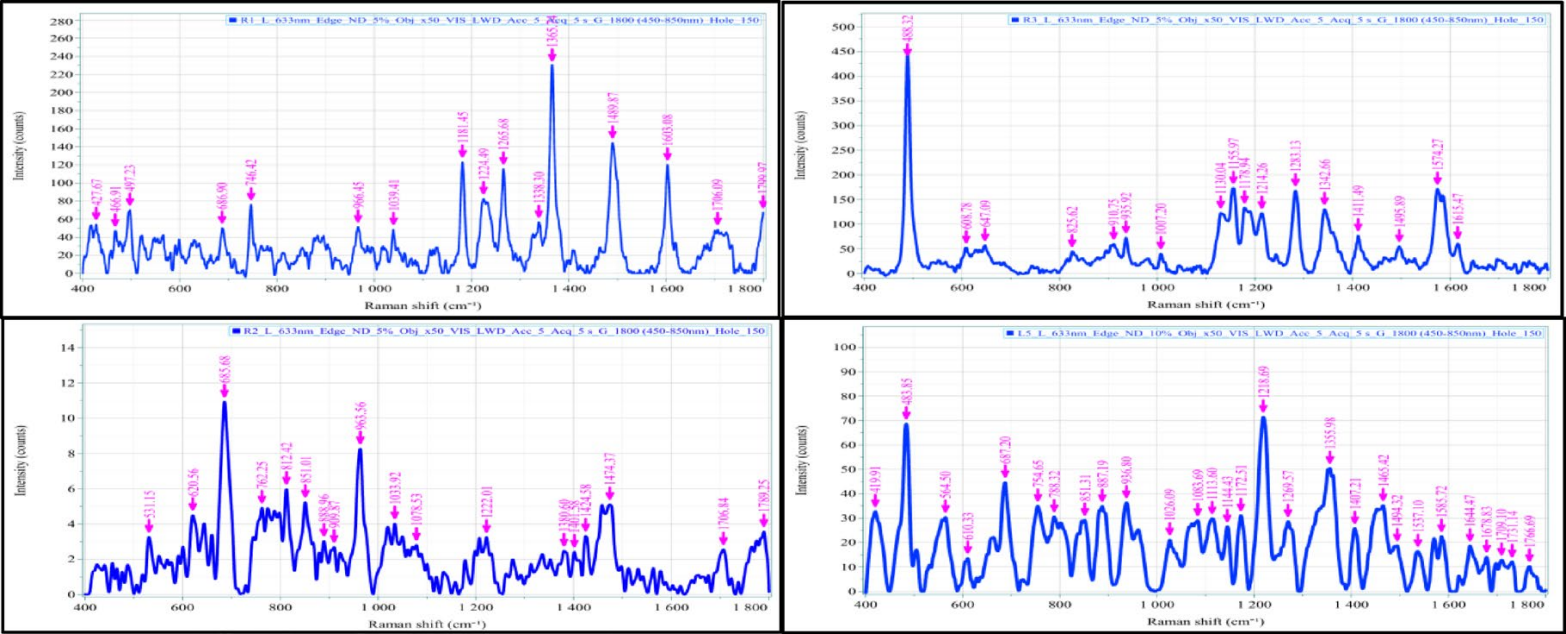
Raman spectra of the selected microplastics.

These findings confirmed that environmental aging and exposure had altered the sample, with degradation products likely influencing the surface composition. This elemental profile aligned with common types of ecological microplastics found in marine and terrestrial settings, illustrating the complex interplay of degradation, contamination, and polymer identity^35,37,45,65–67^. Following the visual inspection results, one microplastic particles were selected for polymer identification using a Raman micro-spectrophotometer (Fig. 7). The Raman analysis provides further confirmation of the material composition, displaying distinct peaks corresponding to the molecular vibrations. The Open Specy library was utilized to identify the polymers in the microplastics (MPs) samples^68^. Only polymers with a matching accuracy exceeding 70. % compared to the reference polymers were accepted (Fig. 7). The obtained Raman spectra confirmed the presence of PE, PP, and PET, which are consistent with the dominant polymer types identified through SEM/EDS analysis. These polymers are also among the most frequently reported in previous studies conducted in the Persian Gulf, supporting the representativeness of our findings. A detailed comparison of the characteristics of microplastics found in water, sediment, and fish samples from this study area versus those in other regions can be found in Table 2.

**Table 2:** Comparison of the characteristics of microplastics in water, sediment, and fish between this study area and other regions.

Region of samples	Sample type	Prevail color	Prevail shapes	Prevail Size	Prevail polymers	References
Persian Gulf, Iran	Water, Fish, and Sediment	Black/Gray	Fiber	≤ 250 μm	N/I	This study
Persian Gulf, Iran	Sediment	White	N/I	< 0.25 mm	N/I	^23^
Persian Gulf, Iran	Ballast water Seawater	Black/Gray	Fiber	50–300 μm	PC	^32^
Persian Gulf, Iran	Fish	Black/Gray	Fibrous fragment	100–250 μm	N/I	^78^
Persian Gulf, Iran	Aquatic species	Black/Gray	Fiber	≥ 1000 μm	PET, PP, HDPE, PS, and NYL	^37^
Persian Gulf, Iran	Water, Fish, and Sediment	Black	Fiber	0.3 to 5 mm	PP, PE	^45^
Persian Gulf, Iran	Water, and Sediment	White	Fragment and foam	1–4.75 mm	PE, PP, and PS	^65^
Caspian Sea, Iran	Sediment Water	Black	Fibre	Sediment: 250–500 μm Water: >1000 μm	PET, PS, and NYL	^35^
Anzali Wetland, Iran,	Sediment Water	Blue/Green	Fiber	50–5000 μm	PP	^67^
Hashilan Wetland, Iran	Sediment Water	Sediment: Black/Gray Water: Blue/Green	Fiber	Sediment: <100 μm Water: <100 μm	Sediment: PP Water: PP	^66^
Oslofjord, Norway	Sediment	Transparent	Fibre	30–600 μm	PS, PP	^33^
Gulf of Thailand	Water		Fiber and fragment	125–300 μm	PP, PE	^79^
Baru and Trisik Beaches, Indonesia	Seawater Fish	Black	Film	< 1.5 mm	PP, PE, and PET	^80^
Thermaic Gulf, North Aegean Sea	Water, Fish, and Sediment	White	Fragment	1–5 mm	PP, PE	^81^
Tanchon stream, Korea	Water, Fish, and Sediment	N/I	Fragment	0.1–0.3 mm	PE, PP	^82^
Alappuzha, India	Water, Fish, and Sediment	White	Fragment	Water: 1–5 mm Sediments: <1 mm	PP, PS, PA, and PE	^83^
Qatar Coast	Beach, Sediment	Black	Pellets	3000–4000 μm	PP, PE	^84^
Abu Dhabi Emirate, UAE	Water and Sediment	Red, Transparent	Filaments	100–300 μm	ABS, PA66, CA, PET	^85^
Khor Al-Zubair, NW Arabian Gulf, Iraq	Benthic Sediment	Black	Fragments, Fibers	0.3–1.2 mm	PE, PP	^86^

N/I: Not Identified; PP: Polypropylene, PS: Polystyrene, PE: Polyethylene, .

HDPE: High-density polyethylene, PC: Polycarbonate, PET: Polyethylene Terephthalate, NYL: Nylon.

### Ecological risk assessment

**Fig. 8:**
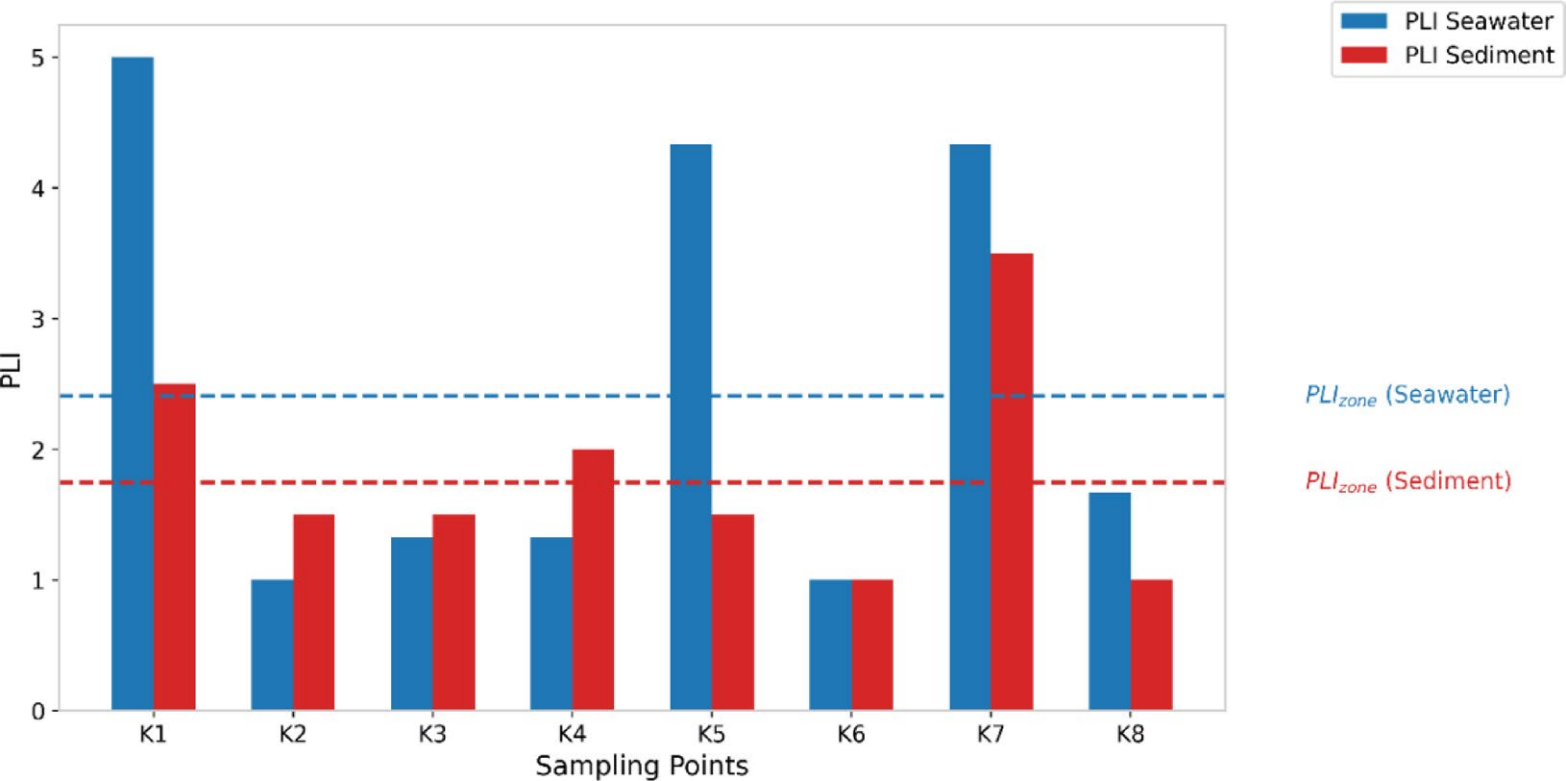
Ecological risk assessment of MPs across zones using PLI and PLI_Zone_ in seawater and sediment.

The biological risk posed by microplastic contamination in seawater and sediment across the eight sampling zones (K1–K8) was assessed using the Pollution Load Index (PLI). PLI values were calculated for each zone relative to baseline concentrations (C_0_ = 3 items L⁻¹ for seawater and C_0_ = 10 items kg⁻¹ for sediment). In seawater, PLI values ranged from 1 in K2 and K6 to 5 in K1, indicating moderate to high contamination in zones K1, K5, and K7. Sediment PLI values varied between 1 (K6 and K8) and 3.5 (K7), with the highest contamination observed in K7. The overall PLI across all zones was 2.41 for seawater and 1.75 for sediment, reflecting a moderate ecological risk in the water column and a lower but non-negligible risk in sediments (Fig. 8). These results demonstrated that microplastic pollution was more pronounced in the water column, although hotspots such as K7 exhibited elevated contamination in both water and sediment. Zones with PLI values exceeding 2 (K1, K5, and K7 for seawater) were classified as areas of moderate to high ecological risk, where microplastic accumulation could potentially affect filter-feeding organisms and benthic communities. Sediment-associated risks were generally lower, yet elevated PLI in K7 indicated localized hotspots that might impact sediment-dwelling organisms and trophic transfer. The spatial distribution of PLI highlighted a heterogeneous pattern of microplastic contamination, emphasizing the importance of implementing targeted mitigation strategies in high-risk zones. These observations were consistent with previous studies reporting that higher microplastic concentrations are associated with increased biological stress in aquatic ecosystems^69,70^.

### Spatial analysis

**Fig. 9:**
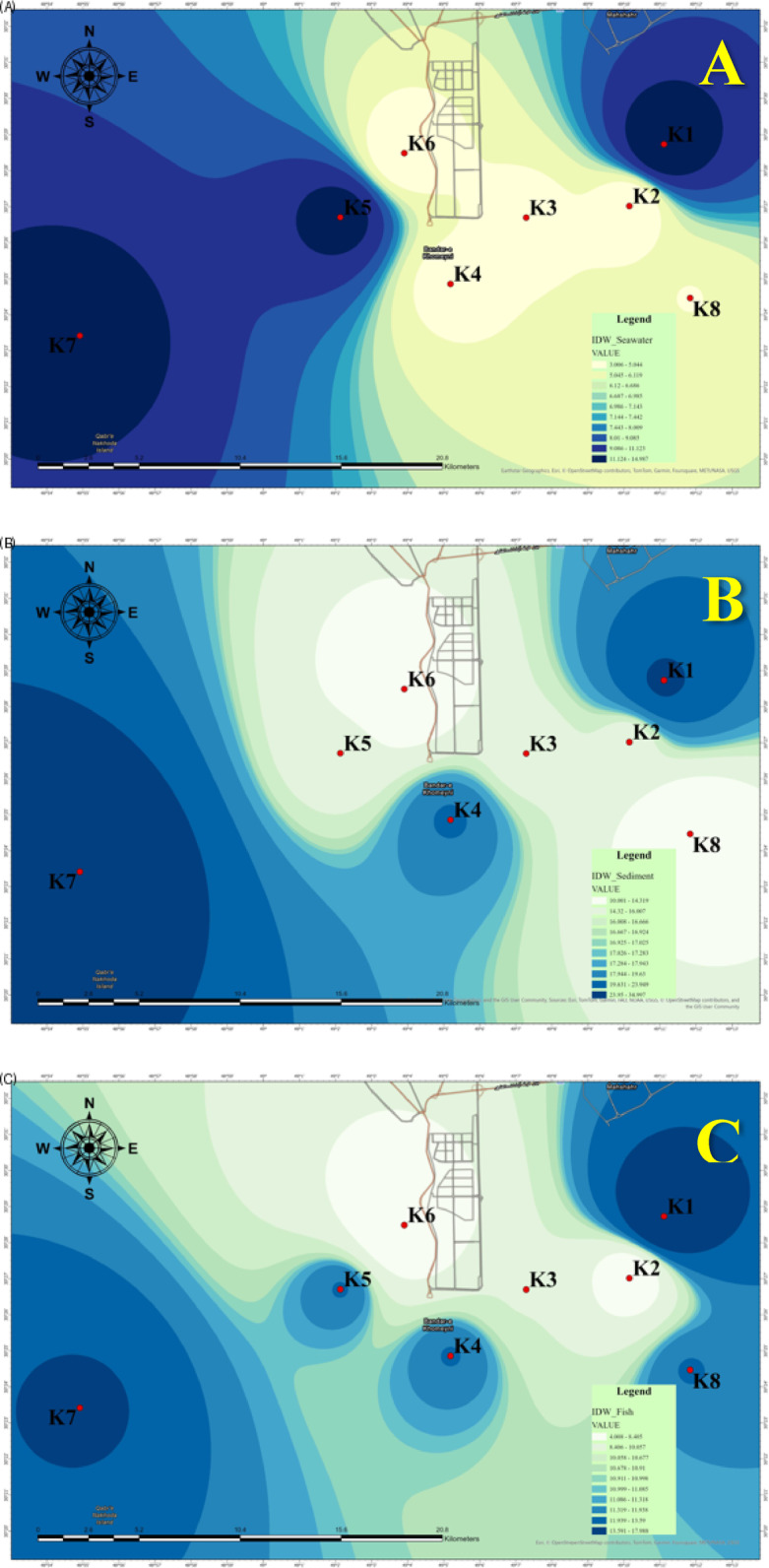
Spatial distribution map of MPs in (**a**) seawater, (**b**) sediment, and (**c**) fish samples using IDW interpolation.

Figure 9 illustrates the spatial distribution of microplastics (MPs) in seawater, sediment, and fish samples, employing Inverse Distance Weighting (IDW) interpolation. The highest concentration of MPs in seawater was detected at K1 (Khor Semaili), measuring 15 items/L, while the lowest concentrations were noted at K2 and K6, each with 3 items/L. The IDW analysis identified significant pollution hotspots at Khor Semaili and Douragh (K5), with MPs concentrations gradually declining towards southern sites, such as K7 (Khor Bihad). This spatial distribution indicates the potential influence of localized pollution sources or limited water circulation in the northern regions.

In sediment samples, K7 (Khor Bihad) exhibited the highest MPs concentration at 35 items/Kg, suggesting that contamination is more severe in southern estuaries. In contrast, K6 (Khor Zangi) and K8 (Khor Ghazaleh) showed lower MPs counts, likely due to site-specific factors, including sediment composition and reduced deposition rates.

For fish samples, the highest MPs concentration was also recorded at K1 (Khor Semaili), with 18 items/10 g. The IDW interpolation revealed a decreasing concentration gradient toward the southwest, with minimal accumulation at K2 and K6. This distribution pattern indicates that fish in northern regions are at a heightened risk of MPs ingestion, likely attributable to elevated contamination levels in adjacent waters and localized MPs accumulation.

This comprehensive analysis underscores the spatial variability of MPs pollution across various environmental matrices, highlighting the urgent need for targeted mitigation strategies in hotspots like Khor Semaili (K1) and the southern estuaries. The IDW method effectively visualizes localized variations, demonstrating that the northern site (K1) is a significant MPs hotspot, while areas such as K6 and K8 show minimal contamination across all matrices (water, sediment, and fish).

The spatial analysis of microplastics (MPs) distribution across seawater, sediment, and fish samples reveals critical insights into pollution dynamics within the studied marine environment.

Aghadadashi et al. conducted a comprehensive basin-wide study employing GIS-based techniques to evaluate the levels, spatial behaviors, and potential risks associated with microplastics and phthalates. They utilized methodologies such as semi-variograms and trend analysis to identify spatial outliers and major deposition sites. Additionally, they identified a risk hotspot in the Sea of Makran, covering approximately 342.99 km², using fuzzy logic and GIS algorithms^28^. Similarly, Tatan et al. investigated 30 groundwater boreholes in the Rahmaniya, Bedee, and Falah areas of Sharjah, UAE. Their GIS analysis, which utilized IDW interpolation, revealed significant contamination from microplastics in the Rahmaniya region. The study also identified potential contamination sources, including industrial zones, the Sajaa landfill, Bedee Farmland, and the water disposal lagoon^71^.

The IDW interpolation indicates that Khor Semaili (K1) is a significant hotspot for MP contamination. This elevated presence suggests localized pollution sources, potentially exacerbated by limited water circulation in the northern regions. Such patterns emphasize the need for focused mitigation efforts to address pollution sources in this area, including monitoring land-based activities and industrial discharges. Comparable spatial patterns of MP accumulation have been reported in other regional aquatic systems, such as the Ganga–Brahmaputra delta and the Himalayan freshwater lakes, where localized industrial and urban discharges were identified as dominant sources of contamination^72,73^.

Conversely, lower concentrations at sites like K2 and K6 reflect spatial variability influenced by local factors, such as hydrodynamics and sediment characteristics.

Furthermore, the findings reveal that fish populations in northern locations are at a heightened risk of MP ingestion. This risk is compounded by the identified spatial concentration gradient, highlighting how environmental matrices interact and contribute to bioaccumulation processes. These results underscore the importance of comprehensive monitoring across various matrices to better understand the implications of MPs pollution on marine ecosystems. Targeted management strategies should prioritize hotspot areas like Khor Semaili and southern estuaries, which could significantly mitigate the impact of MPs contamination on marine life and water quality.

Moreover, the observed spatial distribution patterns highlight the role of local waste management practices in influencing microplastic pollution. In the study area, municipal solid waste is largely collected and transported to landfills with minimal source segregation, and recycling initiatives are limited. Coastal activities, including fishing and port operations, further contribute to plastic debris, exacerbating contamination in identified hotspots. To mitigate microplastic inputs, improvements in waste management are crucial. Recommended measures include implementing source segregation programs, expanding community-based recycling initiatives targeting plastic waste, enforcing regulations on plastic disposal from fisheries and industrial activities, and conducting public awareness campaigns emphasizing the ecological consequences of plastic pollution. Such interventions, coupled with continued monitoring, are likely to reduce microplastic accumulation in critical hotspots like Khor Semaili (K1)^74–77^.

## Conclusions

This study provides essential insights into the spatial distribution of MPs within the marine environments of the Persian Gulf, highlighting an emerging environmental crisis with significant implications for marine ecosystems and public health. Our comprehensive analysis reveals that microplastics are pervasive across all examined matrices, including seawater, sediments, and fish, with concentrations indicating a troubling level of contamination. Notably, microplastics were detected in every sample, predominantly in the form of fibers.

The Inverse Distance Weighting (IDW) analysis identified Khor Semaili (K1) as a critical hotspot for microplastic contamination, particularly in seawater and fish samples. In contrast, sites such as Khor Zangi (K6) and Khor Ghazaleh (K8) showed minimal contamination, underscoring the spatial variability and potential influence of localized pollution sources.

Furthermore, strong positive correlations were observed between microplastic contamination in seawater and fish, as well as between sediments and fish. These findings suggest a direct impact of microplastic pollution on marine life, raising concerns about the potential for bioaccumulation. The elevated concentrations of microplastics found in fish from contaminated hotspots not only threaten marine organisms but also pose risks to human health through seafood consumption.

In light of these findings, policymakers are encouraged to prioritize targeted mitigation strategies, particularly in identified hotspots like Khor Semaili (K1). Recommended actions include implementing regulatory measures to reduce plastic waste discharge, enhancing monitoring of microplastic levels, and launching public awareness campaigns aimed at minimizing plastic pollution at the source.

Collaborative efforts among stakeholders—including government agencies, local communities, and researchers—are crucial to addressing this urgent environmental challenge and safeguarding both marine ecosystems and public health in the Gulf region.

Ultimately, this study highlights the urgent need for ongoing research and decisive action to combat the pervasive threat of microplastics in marine environments, thereby ensuring the health of both marine biodiversity and the communities that rely on these vital ecosystems.

## Limitations and future work

This study offers important insights into the spatial distribution of microplastics (MPs) across seawater, sediments, and fish; however, several limitations should be acknowledged. The assessment did not include a quantitative evaluation of ecological or human health risks associated with microplastic ingestion by marine organisms. As a result, the implications for bioaccumulation, trophic transfer, and seafood safety remain uncertain. In addition, the sampling campaign was limited to a single season and focused on particles larger than 50 μm, potentially underestimating the contribution of smaller micro- and nanoplastics. Future research should therefore incorporate multi-seasonal monitoring, advanced analytical methods capable of detecting submicron particles, and comprehensive risk assessments to better elucidate exposure pathways and their possible effects on marine ecosystems and human health.

## Data Availability

All data are available from the corresponding author upon reasonable request.
